# Morphology and buoyancy-related data for *Posidonia oceanica* fruits

**DOI:** 10.1016/j.dib.2026.113351

**Published:** 2026-10-14

**Authors:** Trace Laskey, Carlo Pipitone, Arturo Zenone, Adriana Alagna, Giovanni D’Anna, Vincenzo Maximiliano Giacalone, Eduardo Infantes, Marco Martinez, Fabio Badalamenti

**Affiliations:** a School of Geosciences, University of Edinburgh, The King’s Buildings, Edinburgh EH9 3FE, UK; b Institute for marine biological resources and biotechnology, IRBIM-CNR, Palermo, Italy; c Sicily Marine Centre, Stazione Zoologica Anton Dohrn, Palermo, Italy; d National Biodiversity Future Centre (NBFC), Palermo, Italy; e Institute for marine biological resources and biotechnology, IRBIM-CNR, Castellammare del Golfo, Italy; f Institute for marine biological resources and biotechnology, IRBIM-CNR, Campobello di Mazara, Italy; g Department of Biology, Campus Tafira, University of Las Palmas de Gran Canaria, Las Palmas, Canary Islands, Spain; h Italian Institute for Environmental Protection and Research, ISPRA, Rome, Italy

**Keywords:** Seagrass, Morphometric traits, Marine angiosperm, Mediterranean Sea: plant reproductive biology

## Abstract

This article presents a dataset on the morphology- and buoyancy-related measurements of *Posidonia oceanica* fruits. The dataset includes direct measurements of fruit mass, three primary axes, volume, dimensions of the exposed portion of floating fruits, and dorsal protrusion height. In addition to measured variables, the dataset contains derived morphology data via previously measured values and established estimation equations describing fruit submerged and emerged volume when floating, density, total surface area, and exposed surface area when floating. These data provide a resource for analyses of fruit size, shape, density, and buoyancy in *P. oceanica*. The dataset may be reused in morphometric analyses and trait-based comparisons. It may also provide empirical trait values for future experimental and modelling studies of near-surface fruit dispersal.

Specifications Table

**Table:** 

Subject	Biology
Specific subject area	Marine botany/biology Morphological measurements and estimations of *Posidonia oceanica* fruit features.
Type of data	Raw measurements and observations, together with geometry-derived estimates, physics-based estimates, calculated variables, and summary variables derived from repeated observations.
Data collection	Data were collected using various laboratory equipment including callipers (precision 0.1 mm) for length measurements, analytical balance (precision 0.0001 g) for mass measurements, and a graduated cylinder with 1 mL graduations for displacement-based volume measurements; water levels were photographed before and after fruit submersion and quantified by image-based interpolation in ImageJ 1.54 (U.S. National Institutes of Health, Bethesda, MD, USA), using a 2 mL interval of the visible graduation scale for calibration. Derived variables were obtained from the primary measurements using geometric approximations for fruit and protrusion volume and surface area, arithmetic calculations for density and related variables, and Archimedes’ principle for submerged volume.
Data source location	Isola delle Femmine, Gulf of Carini, north-western Sicily, Italy (38°11′ N, 13°14′ E). Data are stored at IRBIM-CNR – Institute for marine biological resources and biotechnology, Palermo, Italy.
Data accessibility	Repository name: *Posidonia oceanica* fruits measurements Data identification number: DOI: https://doi.org/10.5281/zenodo.22641465 Direct URL to data: https://zenodo.org/records/22641465 Instructions for accessing these data: The dataset and associated files are openly accessible through the Zenodo repository
Related research article	T. Laskey, A. Zenone, A. Alagna, G. D’Anna, V.M. Giacalone, E. Infantes, M. Martinez, C. Pipitone, F. Badalamenti, Fruit morphology of *Posidonia oceanica* (L.) Delile and its relationship to floating orientation and potential surface dispersal, Aquat. Bot. 207 (2026) 104047. https://doi.org/10.1016/j.aquabot.2026.104047

## 1 Value of the Data

• This dataset provides individual-level morphological and buoyancy-related measurements for *Posidonia oceanica* fruits collected during the 2025 reproductive season at one location. By combining direct fruit measurements with derived geometric approximations, the dataset provides a structured resource for characterizing multiple physical traits of seagrass fruits.
• The data are relevant to researchers working on seagrass reproductive ecology, fruit and propagule traits and comparative morphology of marine angiosperms. The combination of standard morphometric descriptors with flotation-related variables enables comparisons among fruit traits and among alternative approaches for estimating volume, density, surface area, and submerged or emerged fractions.
• The data may also support future studies of seagrass fruit dispersal by providing empirical trait values related to fruit morphology, density, floating orientation, and the proportion of the fruit exposed above the water surface. These measurements can be used as input parameters or reference values in future experimental and modelling studies of near-surface dispersal.

## 2 Background

This data article supports the associated research article [1] and describes the dataset deposited in the linked repository [2]. Seagrass dispersal and connectivity depend partly on the physical properties of reproductive propagules, including size, buoyancy, and interaction with the air–sea interface during floating transport [3,4]. In *Posidonia oceanica*, previous studies have described sexual reproduction, seed and early plantlet traits, fruit-mediated dispersal, and the viability of stranded fruits [[5], [6], [7], [8]]. Fruits can remain buoyant at the sea surface before seed release, making fruit morphology and flotation-related traits relevant to the description of this dispersal phase [5,7,8]. However, quantitative datasets that combine fruit size, mass, volume, density, and derived estimates of emerged and submerged fractions remain limited for this species. Recent modelling studies further highlight the importance of propagule traits for predicting dispersal and connectivity [[9], [10], [11]], while trait-based frameworks emphasise the value of comparable morphological and functional measurements for seagrass ecology [12]. Hydrodynamic processes and propagule behaviour can further influence dispersal outcomes, reinforcing the value of empirical datasets describing fruit morphology and flotation-related traits [13].

This dataset was compiled to document morphological and buoyancy-related traits of *P. oceanica* fruits collected from a Mediterranean beach-cast accumulation event and to provide both measured and derived descriptors that can be compared across studies. The data include direct morphometric measurements, displacement-based volume measurements, and protrusion-based flotation-related measurements, together with variables estimated from geometric approximations and Archimedes-based calculations. The dataset was generated in the context of a broader investigation of fruit form, floating orientation, and exposed surface characteristics during the dispersal phase, and provides the underlying measurements and derived variables used to describe these properties at the individual fruit level.

## 3 Data Description

This article describes the *Posidonia oceanica* fruit morphology dataset deposited in the linked repository [2]. Fruits were collected over three consecutive days during the 2025 reproductive season. The restricted collection window was chosen to maximise fruit freshness and minimise variability associated with differences in post-detachment and beach-exposure time. Consequently, the dataset is intended to characterise this specific fruit cohort and should not be interpreted as representing geographic, seasonal or population-level variability across *P. oceanica*. The dataset contains fruit-level measurements and derived variables describing morphology, mass, volume, density, and flotation-related characteristics of *P. oceanica* fruits collected at Isola delle Femmine, Gulf of Carini, north-western Sicily, Italy, in April 2025. In total, 196 fruits were collected, of which 100 were included in the morphology dataset described here. Variable availability follows the hierarchical sampling design: basic morphometric measurements and wet mass are available for all 100 fruits, displacement-based volume measurements for 41 fruits, and dorsal protrusion measurements and flotation-orientation data for 18 of those 41 fruits. Blank cells indicate variables that were not measured or were not applicable under this design and do not represent zero values or data loss.

The deposited spreadsheet contains two sheets: morphology_data, including the main fruit-level morphometric and buoyancy-related variables for 100 fruits, and flotation orientation trials, including the 20 repeated flotation-orientation trials for the 18-fruit flotation subset. The morphology_data sheet is organised at the level of individual fruits and uses a unique fruit ID for each observation. It includes directly measured variables, such as fruit wet mass, three primary axes, displacement-based volume, dorsal protrusion diameters, and dorsal protrusion height, together with variables derived from geometric approximations and Archimedes-based calculations. The flotation_orientation_trials sheet reports the binary outcome of 20 repeated flotation trials for each of the 18 fruits used in the flotation subset, together with fruit-level counts and frequencies of dorsal-protrusion-up and other orientations. The origin of each variable and, for derived variables, its dependencies on underlying measurements are documented in the accompanying data dictionary. Derived variables sharing the same underlying measurements are mathematically related and should not be treated as independent measurements.

Variables in the dataset are explicitly classified according to their origin as direct measurements or observations, geometry-derived estimates, physics-based estimates, calculated variables, or summary variables derived from repeated observations. This distinction is reported in the accompanying data dictionary to make dependencies among variables transparent. The repository also includes an accompanying data dictionary, a README file describing dataset structure and quality-control procedures, the R script used for the revised analyses, and the corresponding analysis output.

An overview of the variables included in both sheets of the deposited spreadsheet is provided in Table 1.

**Table 1:** Overview of variables included in the deposited dataset.

Sheet	Variable	Description	Unit	n fruits
morphology_data	fruit_id	Unique identifier for each fruit	–	100
morphology_data	wet_mass_g	Fruit wet mass after gentle blotting	g	100
morphology_data	axis_major_cm	Major fruit axis, corresponding to maximum fruit length, measured with a digital calliper	cm	100
morphology_data	axis_intermediate_cm	Intermediate fruit axis, corresponding to maximum fruit width, measured with a digital calliper	cm	100
morphology_data	axis_minor_cm	Minor fruit axis, corresponding to maximum fruit thickness, measured with a digital calliper	cm	100
morphology_data	volume_estimated_cm3	Fruit volume estimated from the triaxial ellipsoid approximation	cm³	100
morphology_data	density_estimated_g_cm3	Fruit density calculated from wet mass and estimated volume	g cm⁻³	100
morphology_data	surface_area_estimated_cm2	Total fruit surface area estimated using Knud Thomsen’s approximation for a triaxial ellipsoid	cm²	100
morphology_data	wet_mass_after_marking_g	Fruit wet mass after application of the small marker dot used for flotation trials	g	18
morphology_data	volume_displacement_cm3	Fruit volume measured by seawater displacement	cm³	41
morphology_data	protrusion_minor_diameter_cm	Minor diameter of the exposed dorsal protrusion measured after excision	cm	18
morphology_data	protrusion_major_diameter_cm	Major diameter of the exposed dorsal protrusion measured after excision	cm	18
morphology_data	surface_area_emerged_cm2	Surface area of the emerged dorsal protrusion estimated by approximating it as the upper half of a triaxial ellipsoid using Knud Thomsen’s approximation	cm²	18
morphology_data	protrusion_height_cm	Maximum height of the exposed dorsal protrusion	cm	18
morphology_data	volume_emerged_ protrusion_cm3	Emerged volume estimated from dorsal protrusion geometry using an ellipsoidal dome approximation	cm³	18
morphology_data	density_displacement_g_cm3	Fruit density calculated from wet mass and displacement-based volume	g cm⁻³	41
morphology_data	volume_submerged_ protrusion_cm3	Submerged volume calculated as displacement-based total volume minus protrusion-derived emerged volume	cm³	18
morphology_data	volume_submerged_ archimedes_cm3	Submerged fruit volume estimated from wet mass using Archimedes’ principle	cm³	100
flotation_orientation_trials	fruit_id	Unique identifier matching the corresponding fruit in the morphology_data sheet	–	18
flotation_orientation_trials	orientation_trial_ 1–orientation_trial_20	Binary flotation outcome for each of 20 repeated flotation trials; 1 = dorsal protrusion facing upward, 0 = other orientation.	0/1	18
flotation_orientation_trials	n_dorsal_protrusion_up	Number of trials in which the dorsal protrusion faced upward	count	18
flotation_orientation_trials	n_other_orientation	Number of trials in which another orientation occurred	count	18
flotation_orientation_trials	expected_dorsal_protrusion_up	Expected number of dorsal-up outcomes under a 0.5 null expectation	count	18
flotation_orientation_trials	frequency_dorsal_ protrusion_up_percent	Percentage of trials in which the dorsal protrusion faced upward	%	18
flotation_orientation_trials	frequency_other_ orientation_percent	Percentage of trials in which another orientation occurred	%	18

## 4 Experimental Design, Materials and Methods

### 4.1 Sample collection and handling

*Posidonia oceanica* fruits were collected along the coast of Isola delle Femmine (Gulf of Carini, north-western Sicily, central Mediterranean; 38°11′ N, 13°14′ E) between 21 and 23 April 2025. Sampling was conducted on three consecutive mornings at the tideline during the onset of the high-tide phase following strong north-westerly winds that are known to facilitate the stranding of floating materials in the area. Fruits were collected manually from beach-cast material and selected based on external freshness criteria, including intact pedicels, attached dark green sepals, turgid pericarp tissue, and the absence of visible dehiscence. The short sampling window was intended to maximize fruit freshness and to minimize variation associated with post-detachment and beach exposure, although the exact time elapsed since detachment was unknown.

A total of 196 fruits were collected. Of these, 100 fruits were allocated to the morphology dataset and 96 fruits to dehiscence observations. The morphology subset was measured using a hierarchical workflow: all 100 fruits were used for basic morphometric measurements and wet mass, 41 fruits were additionally used for displacement-based volume measurements, and 18 fruits out of these 41 were further used for flotation trials and dorsal protrusion measurements. Fruits were transported in cooled seawater to the laboratory and stored in seawater at 4 °C and 38‰ salinity until processing. Fruits included in the morphology dataset were processed within 48 h of collection. Seawater was renewed regularly during storage.

### 4.2 Fruit morphology and mass

The major, intermediate, and minor axes of all 100 fruits were measured using a digital caliper with a precision of 0.1 mm. Axes were defined as the maximum fruit length, width, and thickness, respectively, and were recorded in centimeters. Following axial measurements, fruits were gently blotted with absorbent paper for approximately 10 s to remove excess surface water and were then weighed on an analytical balance (precision ±0.0001 g). Wet mass was recorded in grams.

### 4.3 Displacement-based volume

After completion of axial and wet mass measurements, a target of approximately 40 fruits was set for displacement-based volume measurements. Fruits were randomly selected from the 100-fruit morphology subset after excluding individuals showing visible external damage, tissue loss, or other alterations that could affect volume measurements. Forty-one eligible fruits were ultimately selected, and all valid measurements were retained. Fruit volume was determined by seawater displacement using a vertically positioned graduated cylinder with 1 mL graduations. Each fruit was fully submerged using a thin wooden dowel. For each measurement, the water level was photographed immediately before and after complete fruit submersion. Images were analysed in ImageJ using a 2 mL interval of the visible graduation scale for calibration. Water level was determined from the central portion of the meniscus, and fruit volume was calculated as the difference between the post- and pre-immersion levels. Image-based interpolation allowed water-level changes falling between adjacent graduations to be quantified; consequently, the decimal places reported in the dataset reflect image-analysis output rather than the nominal accuracy of the graduated cylinder. Displacement-based fruit volumes ranged from 1.30 to 3.30 cm³ across the 41 measured fruits.

The complete displacement procedure was not systematically replicated for every fruit. When individual readings were unclear, additional photographs and/or repeated measurements were obtained as needed. These repeats were used to obtain a clearer estimate rather than as part of a predefined repeatability experiment. Prior control measurements, documented photographically and evaluated using the same image-analysis procedure, showed that inserting the thin dowel to the depth used during fruit measurements did not produce a detectable change in water level.

The remaining 59 fruits were retained without further experimental manipulation, frozen for long-term storage, and reserved for future analyses outside the scope of the present dataset.

### 4.4 Estimates of fruit volume, density, and surface area

Fruit volume and surface-related variables were derived using geometric approximations commonly applied to fruit shape and ellipsoid-like objects [[14], [15], [16]]. These approximations were used as standardised descriptors rather than as exact representations of fruit geometry. For all 100 fruits, total fruit volume was estimated from the three axial measurements by approximating each fruit as a triaxial ellipsoid. Estimated volume was calculated as:

V=(π/6)×a×b×c

where a, b, and c are the major, intermediate, and minor axes, respectively.

Fruit density was calculated as wet mass divided by fruit volume. Two density variables were produced: density based on estimated volume and density based on displacement volume. Density was calculated as:

ρ=m/V

where m is fruit wet mass in grams and V is fruit volume in cm³.

Total fruit surface area was estimated for all fruits using Knud Thomsen’s approximation for the surface area of a triaxial ellipsoid:

$$SA_{\text{total}}\approx 4\pi \times {\left[\left({\left(\left(a/2\right)\times \left(b/2\right)\right)}^{p}+{\left(\left(a/2\right)\times \left(c/2\right)\right)}^{p}+{\left(\left(b/2\right)\times \left(c/2\right)\right)}^{p}\right)/3\right]}^{\left(1/p\right)}$$

where a, b, and c are the major, intermediate, and minor axes, respectively, and p = 1.6075.

For the 41 fruits with displacement-based volume measurements, the ellipsoid approximation showed good agreement with measured volume (R² = 0.866; MAE = 0.153 cm³; RMSE = 0.191 cm³), with a small positive mean bias of 0.086 cm³ (approximately 3.7% of mean measured volume).

### 4.5 Archimedes based estimates

For each fruit, the submerged volume was estimated using Archimedes’ principle. Seawater density was set to 1.027 g/cm³, corresponding to Mediterranean seawater at 38‰ salinity and approximately 20 °C. Submerged volume estimated from fruit mass was calculated as:

$$V_{\text{submerged},\;\text{Arch}}=m/\rho_{\text{seawater}}$$

where m is fruit wet mass in grams and ρ_seawater_ is seawater density.

The calculation assumes static free-floating equilibrium in seawater of the specified density, no contact between the fruit and the container, and negligible contributions from surface tension or trapped air. These assumptions were not independently quantified.

### 4.6 Multiple regression model for fruit mass

A multiple linear regression model was fitted using the 100-fruit morphology subset to evaluate the relationship between measured fruit wet mass and the three measured fruit axes. The fitted model was:

Wetmass=−3.165+0.681a+0.917b+1.655c

where a, b, and c are the major, intermediate, and minor axes, respectively.

Coefficient estimates (±SE) were −3.165 ± 0.174 for the intercept, 0.681 ± 0.056 for the major axis, 0.917 ± 0.237 for the intermediate axis, and 1.655 ± 0.264 for the minor axis; corresponding 95% confidence intervals were −3.510 to −2.820, 0.570 to 0.792, 0.447 to 1.388, and 1.130 to 2.179, respectively.

The model explained 90.2% of the observed variation in wet mass (R² = 0.902; adjusted R² = 0.899; F_3,96_ = 295.0, p < 0.001). Because the model was fitted to the same 100 fruits for which wet mass was measured, the resulting in-sample fitted values were not treated as independent predictions and are not included as a separate dataset variable. Predictive performance was instead assessed by leave-one-out cross-validation (LOOCV), yielding R² = 0.894, RMSE = 0.124 g and MAE = 0.100 g. Residual diagnostics did not indicate clear departures from normality or homoscedasticity (Shapiro–Wilk W = 0.992, p = 0.835; Breusch–Pagan χ² = 2.817, df = 3, p = 0.421). Variance inflation factors were 1.19, 4.99 and 4.88 for the major, intermediate and minor axes, respectively, and the maximum Cook’s distance was 0.109. Additional model diagnostics and complete numerical output are provided in the associated analysis script/output.

### 4.7 Flotation and dorsal protrusion measurements

Eighteen fruits from the 41-fruit displacement subset were randomly selected for flotation trials and dorsal protrusion measurements. This subset size reflected the more intensive nature of the repeated flotation procedure and the destructive excision required for protrusion measurements. Each fruit was subjected to 20 repeated flotation trials under laboratory conditions in seawater at 38‰ salinity and approximately 20 °C. For each trial, the fruit was manually introduced into the water with a firm forward motion at an angle of approximately 45–50° to the water surface, without allowing it to contact the bottom. After the fruit returned to the surface, its floating orientation was recorded as either dorsal protrusion facing upward or another orientation. The fruit was then retrieved with a hand net and the procedure was repeated. The dorsal protrusion side of each fruit was identified prior to the trials and marked with a small waterproof marker dot to facilitate repeated observations. Fruit wet mass was recorded before and after application of the marker to document any change associated with marking. The 20 flotation trials performed for each fruit were repeated within-fruit observations and were not treated as independent biological replicates. The biological sample size was therefore n = 18 fruits. For each fruit, the proportion of trials in which the dorsal protrusion faced upward was calculated across the 20 repeated trials.

After repeated flotation trials, each fruit was floated in its predominant orientation and the boundary of the emerged portion above the waterline was marked. This emerged portion broadly corresponded to the dorsal protrusion. The protruding region was then excised with a scalpel and measured using a digital caliper (precision 0.1 mm). The following variables were recorded for the emerged protrusion: major diameter, minor diameter, and protrusion height.

### 4.8 Exposed portion of fruit

The excised dorsal protrusion was treated using a geometric dome approximation to estimate the volume of the fruit portion exposed above the water surface. Major and minor protrusion diameters were converted to semi-axes and protrusion volume was calculated as:

$$V_{\text{dorsal}\;\text{protrusion}}=\left(\pi h/6\right)\times \left(3a_{\text{prot}}\times b_{\text{prot}}+h^{2}\right)$$

where h is protrusion height and a_prot_ and b_prot_ are the semi-axes corresponding to the major and minor protrusion diameters.

Submerged fruit volume based on protrusion measurements was calculated as total fruit volume minus the exposed protrusion volume. For fruits in the flotation subset, total fruit volume was taken from the displacement-based volume measurement.

The exposed surface area of the dorsal protrusion was estimated by approximating the protrusion as the upper half of a triaxial ellipsoid. Knud Thomsen’s approximation was applied to the corresponding full ellipsoid and the resulting value was divided by two:

$$SA_{\text{emerged}}\approx 2\pi {\left[\left({\left(a_{\text{prot}}\times b_{\text{prot}}\right)}^{p}+{\left(a_{\text{prot}}\times h\right)}^{p}+{\left(b_{\text{prot}}\times h\right)}^{p}\right)/3\right]}^{\left(1/p\right)}$$

where p=1.6075, $a_{\text{prot}}$ and $b_{\text{prot}}$ are the protrusion semi-axes, and h is protrusion height.

No independent direct measurements of emerged volume or emerged surface area were available for validation of the corresponding geometric approximations. These variables should therefore be interpreted as geometry-based estimates derived from directly measured protrusion dimensions.

### 4.9 Data processing

All primary measurements and observations were entered at the individual-fruit level using a unique fruit identifier. Derived variables were then generated from these primary data using several distinct procedures. Geometry-derived estimates were calculated from axial or protrusion measurements using geometric approximations; physics-based estimates were calculated using Archimedes’ principle; and additional calculated variables were obtained through arithmetic combinations of measured or estimated quantities. Summary flotation variables were calculated from the repeated within-fruit orientation observations. These variable origins and their dependencies are identified explicitly in the accompanying data dictionary. Floating orientation was analysed at the fruit level to account for the non-independence of repeated trials. The fruit-specific proportion of dorsal-protrusion-up outcomes was calculated for each of the 18 fruits and compared with the null expectation of 0.5 using a Wilcoxon signed-rank test.

All statistical analyses reported in the revised data article were conducted in R (version 4.5.1) using RStudio (version 2025.09.2 + 418). These included the Wilcoxon signed-rank test, multiple linear regression of fruit wet mass against the three measured fruit axes, internal cross-validation and model diagnostics, and regressions comparing directly measured and estimated variables. Derived variables were calculated from the primary measurements using the equations described above. An accompanying R script recalculates and verifies the deposited derived variables from their documented primary measurements or repeated flotation observations, performs quality-control checks, and reproduces the statistical analyses from the final deposited dataset. The script also performs basic quality-control checks on sample sizes, unique fruit identifiers, subset membership, orientation coding, trial counts, and the expected availability of subset-specific variables.

## Limitations

The dataset is based on beach-cast *Posidonia oceanica* fruits collected from a single location during a three-day sampling period in April 2025. It therefore represents a spatially and temporally restricted cohort and should not be considered representative of geographic or seasonal variation across the Mediterranean. Fruits were selected using external indicators of freshness and absence of visible dehiscence, so visibly dehiscent or deteriorated fruits are not represented. The dataset follows a hierarchical measurement design: basic morphometric measurements are available for all 100 fruits, displacement-based volume measurements for 41 fruits, and flotation-orientation and dorsal protrusion measurements for 18 fruits. Analyses involving the latter variables therefore have smaller biological sample sizes and should be interpreted accordingly. Several variables are derived from geometric or physics-based approximations rather than direct measurement, and their values therefore depend on the assumptions underlying those calculations. Displacement-based volumes were obtained by image-based interpolation of water levels from a graduated cylinder with 1 mL graduations. Although repeated measurements were obtained when individual readings were unclear, systematic replicate measurements were not performed for all fruits; therefore, whole-procedure repeatability and fruit-specific relative measurement error cannot be estimated for the complete displacement subset.

## Ethics Statement

The authors have read and follow the ethical requirements for publication in Data in Brief and confirm that the current work does not involve human subjects, animal experiments, or any data collected from social media platforms.

## Credit Author Statement

TL and FB conceived the study, carried out the measurements and data analyses, and drafted the manuscript. CP assisted with selected measurements. AZ contributed to the estimates based on Archimedes’ principle. AA, MM and AZ contributed to the field experiments on fruit dehiscence. All authors reviewed, revised, and approved the final manuscript.

## Data Availability

ZenodoPosidonia oceanica fruits measurements (Original data)
